# Supplementary material for: Oxic-anoxic regime shifts mediated by feedbacks between biogeochemical processes and microbial community dynamics
Source: Nat Commun. 2017 Oct 6;8:789. doi: 10.1038/s41467-017-00912-x (PMC5630580; doi:10.1038/s41467-017-00912-x)
Supplement: Supplementary file 1 — Supplementary Information [file 41467_2017_912_MOESM1_ESM.pdf]

Supplementary Figure 1. Hysteresis loops for different depths in Lake Vechten. The graphs plot the oxygen saturation level against the inverse of the stratification strength ($1/N^2$, where N^2 is the squared buoyancy frequency). The inverse of the stratification strength provides a simple proxy of oxygen diffusivity across the thermocline (see Methods). A hysteresis loop is found irrespective of whether oxygen saturation is measured at a depth of **a**, 2 m; **b**, 5 m; **c**, 6 m; **d**, 7 m; **e**, 9 m. Data points are from March 2013 to March 2014; arrows indicate the direction of time.

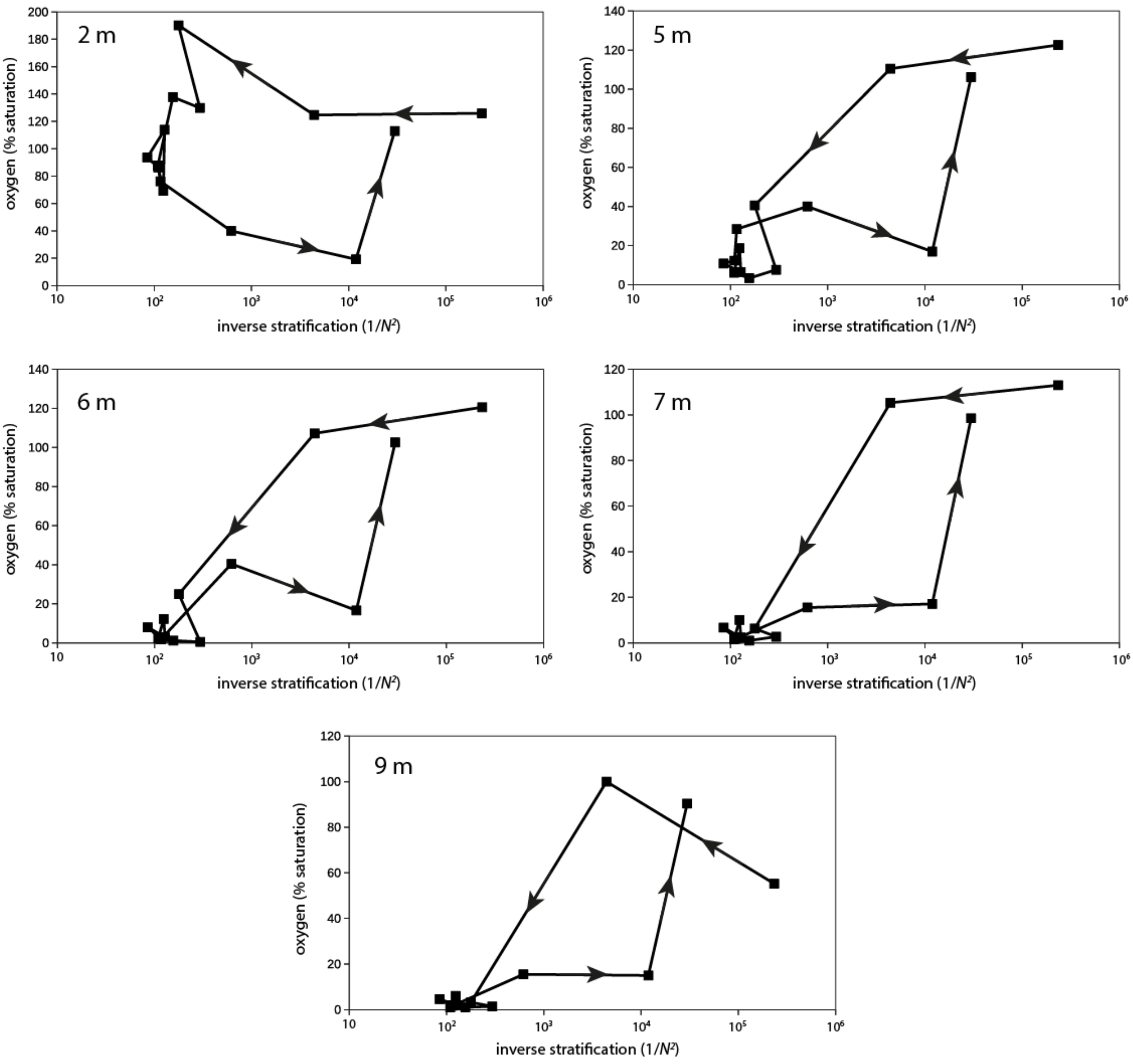

Supplementary Table 1. Parameter values of the model.

Parameter	Meaning	Value	Reference
$g_{\mathrm{max,CB}}$	Maximum specific growth rate of CB	0.05 hr^{-1}	1
$g_{\mathrm{max,PB}}$	Maximum specific growth rate of PB	0.07 hr^{-1}	2
$g_{\mathrm{max,SB}}$	Maximum specific growth rate of SB	0.1 hr^{-1}	3,4
$K_{\mathrm{PB,SR}}$	Half-saturation constant of PB on reduced sulfur	10 μM	5
$K_{\mathrm{SB,SO}}$	Half-saturation constant of SB on oxidized sulfur	5 μM	6
$K_{\mathrm{CB,P}}$	Half-saturation constant of CB on phosphorus	0.2 μM	7
$K_{\mathrm{PB,P}}$	Half-saturation constant of PB on phosphorus	0.5 μM	8
$K_{\mathrm{SB,P}}$	Half-saturation constant of SB on phosphorus	0.5 μM	-
$H_{\mathrm{CB,SR}}$	Half-inhibition constant of CB on reduced sulfur	300 μM	9
$H_{\mathrm{PB,O}}$	Half-inhibition constant of PB on oxygen	100 μM	10
$H_{\mathrm{SB,O}}$	Half-inhibition constant of SB on oxygen	100 μM	10
$y_{\mathrm{SB}}^{\mathrm{SO}}$	Yield of SB on oxidized sulfur	3.33×10^{7} cells $\mu\mathrm{M}^{-1}$	11
$y_{\mathrm{PB}}^{\mathrm{SR}}$	Yield of PB on reduced sulfur	1.25×10^{7} cells $\mu\mathrm{M}^{-1}$	9
$y_{\mathrm{CB}}^{\mathrm{P}}$	Yield of CB on phosphorus	1.67×10^{8} cells $\mu\mathrm{M}^{-1}$	12
$y_{\mathrm{PB}}^{\mathrm{P}}$	Yield of PB on phosphorus	1.67×10^{8} cells $\mu\mathrm{M}^{-1}$	-
$y_{\mathrm{SB}}^{\mathrm{P}}$	Yield of SB on phosphorus	1.67×10^{8} cells $\mu\mathrm{M}^{-1}$	-
p_{CB}	Production of oxygen per cyanobacterial cell	6×10^{-9} μM cell^{-1}	13
m_{CB}	Mortality rate of CB	0.020 hr^{-1}	-
m_{PB}	Mortality rate of PB	0.028 hr^{-1}	-
m_{SB}	Mortality rate of SB	0.040 hr^{-1}	-
α_{S}	Diffusivity of sulfur	0.001 hr^{-1}	-
α_{O}	Diffusivity of oxygen	$10^{-6} - 10^{-2}$ hr^{-1}	-
α_{P}	Diffusivity of phosphorus*	0.01 hr^{-1}	-
$S_{\mathrm{R,b}}$	Background concentration of reduced sulfur	300 μM	14
$S_{\mathrm{O,b}}$	Background concentration of oxidized sulfur	300 μM	14
O_{b}	Background concentration of oxygen	300 μM	15
P_{b}	Background concentration of phosphorus	2 − 10 μM	16
c	Oxidation rate of reduced sulfur	4×10^{-5} $\mu\mathrm{M}^{-1}\,\mathrm{hr}^{-1}$	17,18

CB = cyanobacteria; PB = phototrophic sulfur bacteria; SB = sulfate-reducing bacteria
**We assumed a higher diffusive influx for phosphorus than for sulfur and oxygen, because the phosphorus influx also includes phosphorus release from the sediment and remineralization from dead biomass.*

Supplementary References

1. Paerl, H. W. & Huisman, J. Climate change: a catalyst for global expansion of harmful cyanobacterial blooms. *Env. Microb. Rep.* **1**, 27–37 (2009).
2. Montesinos, E. Change in size of *Chromatium minus* cells in relation to growth rate, sulfur content, and photosynthetic activity: a comparison of pure cultures and field populations. *Appl. Environ. Microbiol.* **53,** 864–871 (1987).

3. Kalyuzhnyi, S., Fedorovich, V., Lens, P., Hulshoff Pol, L. & Lettinga, G. Mathematical modelling as a tool to study population dynamics between sulfate reducing and methanogenic bacteria. *Biodegradation* **9,** 187–199 (1998).
4. Dev, S., Roy, S. & Bhattacharya, J. Understanding the performance of sulfate reducing bacteria based packed bed reactor by growth kinetics study and microbial profiling. *J. Environ. Manag.* **177,** 101–110 (2016).
5. Van Gemerden, H. The sulfide affinity of phototrophic bacteria in relation to the location of elemental sulfur. *Arch. Microbiol.* **139,** 289–294 (1984).
6. Ingvorsen, K., Zehnder, A. J. B. & Jørgensen, B. B. Kinetics of sulfate and acetate uptake by *Desulfobacter postgatei. Appl. Environ. Microbiol.* **47,** 403–408 (1984).
7. Kromkamp, J., Van den Heuvel, A. & Mur, L. R. Phosphorus uptake and photosynthesis by phosphate-limited cultures of the cyanobacterium *Microcystis aeruginosa. Br. Phycol. J.* **24**, 347–355 (1989).
8. Bañeras, L., Ros-Ponsatí, M., Cristina, X. P., Garcia-Gil, J. L. & Borrego, C. M. Phosphorus deficiency and kinetics of alkaline phosphatase in isolates and natural populations of phototrophic sulphur bacteria. *FEMS Microbiol. Ecol.* **73,** 243–253 (2010).
9. De Wit, R., Van den Ende, F. P. & Van Gemerden, H. Mathematical simulation of the interactions among cyanobacteria, purple sulfur bacteria and chemotrophic sulfur bacteria in microbial mat communities. *FEMS Microbiol. Ecol.* **17,** 117–136 (1995).
10. Gerritse, J., Schut, F. & Gottschal, J. C. Modelling of mixed chemostat cultures of an aerobic bacterium, *Comamonas testosteroni*, and an anaerobic bacterium, *Veillonella alcalescens*: comparison with experimental data. *Appl. Environ. Microbiol.* **58,** 1466–1476 (1992).
11. Jin, Q. & Bethke, C. M. The thermodynamics and kinetics of microbial metabolism. *Am. J. Sci.* **307,** 643–677 (2007).
12. Saxton, M. A., Arnold, R. J., Bourbonniere, R. A., McKay, R. M. L. & Wilhelm, S. W. Plasticity of total and intracellular phosphorus quotas in *Microcystis aeruginosa* cultures and Lake Erie algal assemblages. *Front. Microbiol.* **3,** 3 (2012).
13. Gons, H. J. & Rijkeboer, M. The 'true' growth efficiency of phytoplankton as influenced by light attenuation and insolation: implications of the photosynthesis-irradiance relationship. *Hydrobiol.* **238,** 169–176 (1992).
14. Goldhaber, M. B. Sulfur-rich sediments. *Treatise on Geochemistry* **7,** 257–288 (2003).
15. Shaffer, G., Olsen, S. M. & Pedersen, J. O. P. Long-term ocean oxygen depletion in response to carbon dioxide emissions from fossil fuels. *Nat. Geosci.* **2,** 105–109 (2009).
16. Ruttenberg, K. C. The global phosphorus cycle. *Treatise on Geochemistry* **8**, 585–643 (2003).
17. Luther, G. W. *et al.* Thermodynamics and kinetics of sulfide oxidation by oxygen: a look at inorganically controlled reactions and biologically mediated processes in the environment. *Front. Microbiol.* **2,** 62 (2011).
18. Millero, F. J., Hubinger, S., Fernandez, M. & Garnett, S. Oxidation of H_2S in seawater as a function of temperature, pH, and ionic strength. *Environ. Sci. Technol.* **21,** 439–443 (1987).
